# Magnetic‐Assisted Control of Eggs and Embryos via Zona Pellucida‐Linked Nanoparticles

**DOI:** 10.1002/advs.202306901

**Published:** 2024-03-06

**Authors:** Francisco Alberto García‐Vázquez, Gabriela Garrappa, Chiara Luongo, Julieta Gabriela Hamze, María Caballero, Francisco Marco‐Jiménez, José Salvador Vicente Antón, Gregorio J. Molina‐Cuberos, María Jiménez‐Movilla

**Affiliations:** ^1^ Departamento de Fisiología, Facultad de Veterinaria, Campus de Excelencia Mare Nostrum Universidad de Murcia Murcia 30100 Spain; ^2^ Instituto Murciano de Investigación Biosanitaria (IMIB‐Arrixaca) Murcia 30120 Spain; ^3^ Departamento de Biología Celular e Histología, Facultad de Medicina y Enfermería, Campus de Excelencia Mare Nostrum Universidad de Murcia Murcia 30120 Spain; ^4^ Insitituto Nacional de Tecnología Agropecuaria (INTA) Rafaela Santa Fe 2300 Argentina; ^5^ Instituto de Ciencia y Tecnología Animal Universitat Politècnica de València Valencia 46022 Spain; ^6^ Departamento de Electromagnetismo y Electrónica, Facultad de Química Universidad de Murcia Murcia 30100 Spain

**Keywords:** ART, egg, embryo, nanoparticles, zona pellucida

## Abstract

Eggs and embryo manipulation is an important biotechnological challenge to enable positioning, entrapment, and selection of reproductive cells to advance into a new era of nature‐like assisted reproductive technologies. Oviductin (OVGP1) is an abundant protein in the oviduct that binds reversibly to the zona pellucida, an extracellular matrix that surrounds eggs and embryos. Here, the study reports a new method coupling OVGP1 to magnetic nanoparticles (NP) forming a complex (NPOv). NPOv specifically surrounds eggs and embryos in a reversible manner. Eggs/embryos bound to NPOv can be moved or retained when subjected to a magnetic force, and interestingly only mature‐competent eggs are attracted. This procedure is compatible with normal development following gametes function, in vitro fertilization, embryo development and resulting in the birth of healthy offspring. The results provide in vitro proof‐of‐concept that eggs and embryos can be precisely guided in the absence of physical contact by the use of magnets.

## Graphical abstract

Novel method to specifically attach magnetic nanoparticles (NP) through OVGP1 (Ov) protein to the external matrix that surround eggs and embryos. Labeled reproductive cells can be moved or retained in response to a magnetic force while preserving their competence. This methodology demonstrates a robust, non‐toxic technique with the potential to use into a new era of assisted reproductive technologies as a new paradigm.

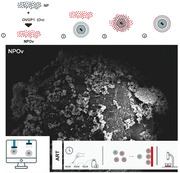

## 1 Introduction

Assisted reproduction technologies (ART) reverse human infertility^[^ ^1^ ^]^ and increase animal reproduction/production in commercial and endangered species.^[^ ^2^, ^3^, ^4^ ^]^ ART require manipulation of gametes and embryos without compromising their ability to fertilize and develop. Since initial successes among mammalian species, there has been constant innovation in protocols for in vitro fertilization (IVF) and in vitro embryo production.

The size and morphology of the mammalian gametes and early embryos make them candidates for on‐chip lab technologies. Novel approaches based on acoustic,^[^ ^5^ ^]^ electric,^[^ ^6^ ^]^ and/or magnetic^[^ ^7^, ^8^, ^9^, ^10^, ^11^, ^12^ ^]^ fields have emerged for handling mammalian gametes and embryos in vitro. In the case of the male gamete, magnetic fields are commonly applied, and in some cases commercially, for sperm cell sorting,^[^ ^11^, ^13^, ^14^ ^]^ semen sexing,^[^ ^15^ ^]^ or for genetic animal modification.^[^ ^16^ ^]^ Conversely, the use of magnetic fields for handling of oocytes/eggs is rare^[^ ^17^, ^18^ ^]^ and has not been reported for embryos.

Nanoparticles (NP) have been used to develop biomedical applications in multiple different areas including diagnostic, treatment or prevention of disease, and medical imaging.^[^ ^19^ ^]^ In reproduction, NP have been mainly applied to male gametes for sperm purification,^[^ ^20^ ^]^ sperm selection,^[^ ^11^, ^13^, ^14^ ^]^ sperm conservation,^[^ ^21^, ^22^, ^23^ ^]^ or as nanocarriers.^[^ ^24^ ^]^ Different approaches have been developed for attachment of NP (or microparticles) to the zona pellucida (ZP) surrounding eggs and embryos^[^ ^25^, ^26^, ^27^ ^]^ or penetrating the ZP.^[^ ^24^, ^28^ ^]^ These methods are being developed for tagging or drug delivery to increase the odds of implantation,^[^ ^29^ ^]^ but not for egg/embryo handling during ART.

Despite their potential utility in biomedicine, modes of administration, dose and duration, size, shape, charge, and composition of surface‐coating of NP raise concerns of potential toxicity.^[^ ^30^ ^]^ On the other hand, the use of NP in ART improves developmental competence of eggs at low NP doses^[^ ^31^, ^32^ ^]^ and increases egg survival after vitrification.^[^ ^33^, ^34^, ^35^ ^]^ Nevertheless, there needs to be an emphasis of safety in exploring interactions of NP and biological reproductive materials (sperm, eggs, or embryos) to improve the efficiency of ART.

Nanotechnology has great potential in reproduction,^[^ ^36^ ^]^ but development of practical applications in ART remains challenging.^[^ ^30^ ^]^ Our group previously reported that recombinant OVGP1 (oviductin‐Ov, an endogenous protein of the oviductal fluid) binds to the surface of the ZP.^[^ ^37^ ^]^ Here we report the development of NP binding to the ZP via the OVGP1 protein (NPOv). This strategy enables efficient binding between the ZP and NP in eggs and embryos with no effect on gametes function or embryo development. We also provide insight into the use of the NPOv complex in ART including vitrification, egg/embryo positioning, and lab‐on‐chip assays.

## 2 Results

### 2.1 Safe Affinity‐Based Technique to Externally Label Eggs and Embryos with Nanoparticles (NP)

**Figure 1:**
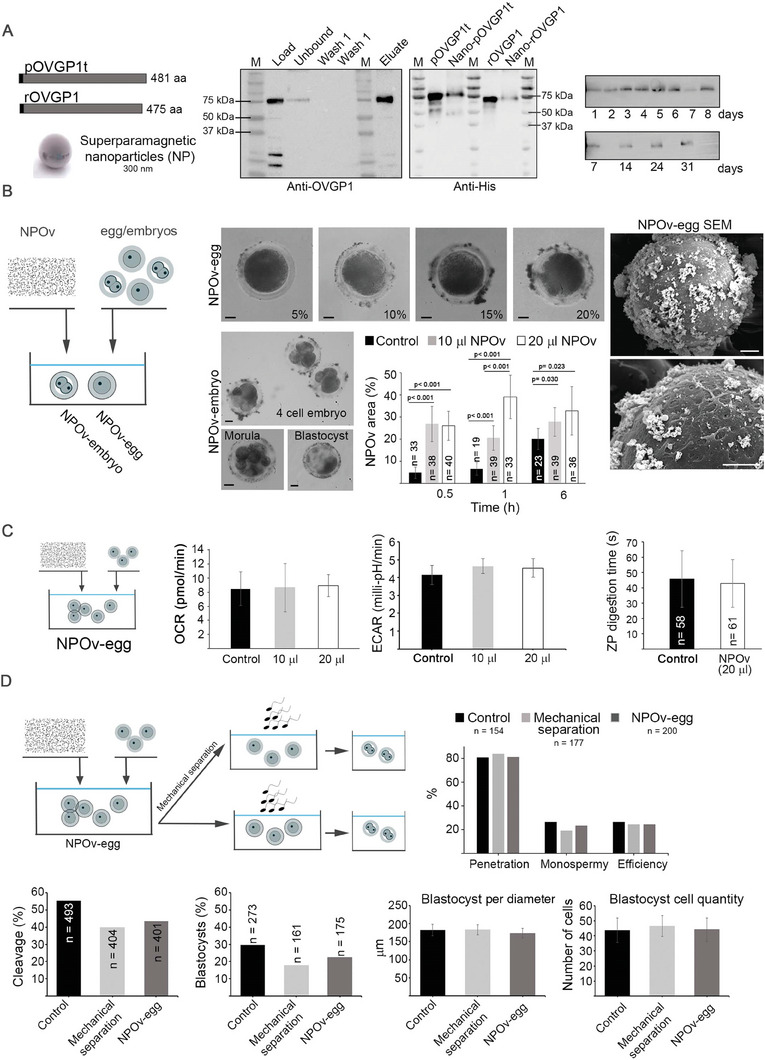
Nanoparticles (NP) conjugated with OVGP1 can attach to the zona pellucida (ZP) of the eggs and embryos without impairing gametes, in vitro fertilization, and embryo development. A) OVGP1 was successfully conjugated to NP. Schematic (left panel) showing truncated porcine OVGP1 (pOVGP1t) and rabbit OVGP1 (rOVGP1) recombinant proteins and NP used in the study. After conjugation of OVGP1 recombinant proteins to NP (NPOv) resultant protein samples were separated by electrophoresis and analysed by western blot (WB) (polyclonal antibody anti‐OVGP1) (left WB). *Load* line indicates pOVGP1t (6 µg; ≈75 kDa); *Unbound* line represents the medium collected after the conjugation of NP. The presence of a 75 kDa band means that not all the protein was conjugated; *Wash 1* and *Wash 2* lines mean the two washes of the NP performed after NPOv conjugation; *Eluate* line is the sample with the only presence of pOVGP1t bound to the NP. The middle WB represents truncated porcine pOVGP1t and rabbit rOVGP1 recombinant proteins before conjugation and the eluted fraction from 20 µL of NP conjugated to pOVGP1t (Nano‐pOVGP1t) and NP conjugated to rOVGP1 (Nano‐rOVGP1). M = molecular marker (kDa). The stability of the NPOv conjugation was evaluated over time (right WBs). Each time point indicates a sample with NP bound to OVGP1 after the days indicated. WBs are representative of experiments repeated three times. B) NPOv are able to attach to the ZP of porcine eggs and embryos. Schematic (left panel) showing NPOv incubation with eggs and embryos. The graph and bright microscope images (middle panels) show the distribution of NPOv around ZP surface (%) which varies between control (NP without OVGP1) and NPOv (10 and 20) groups at each time point (*p* < 0.05). 10 and 20 groups indicate the volume (µL) of NP suspension added to a well of 500 µl. Data are presented as mean ± SEM (*n* = 3 replicates). *Qwin* software (Leica Microsystems Ltd., Barcelona, Spain) was used to evaluate the distribution of NP. Scale bar, 25 µm. Scanning electron microscope (SEM) images (right images) show NPOv (white dots) distributed around ZP of an egg (Scale bar, 25 µm). C) Eggs are not compromised by presence of NPOv. The metabolism of the eggs (oxygen consumption rate‐ORC, pmol/min, and extracellular acidification rate‐ECAR, milli‐pH/min) was similar regardless of the presence and quantity of NPOv (*p* > 0.05). Metabolic measurements were performed with Seahorse XFe96 (Agilent Seahorse analyzer, Agilent Technologies). Data are presented as mean ± SEM (*n* = 3 replicates, 20 eggs per group and replicate). The ZP digestion time was not affected by NPOv presence (U‐Mann Whitney test, *p* > 0.05). Data are presented as mean ± SD. Eggs in the control group were incubated without NPOv. D) In vitro fertilization (IVF) and embryo development were not affected by NPOv. Mature oocytes after incubation with NPOv (and after being removed using mechanical separation by gentle pipetting) were subjected to IVF (*n* = 7 replicates) showing similar output (penetration, monospermy, efficiency; %) between groups (Chi‐Square test, *p* > 0.05). Cleavage (%), blastocyst rate (%), and blastocyst quality (diameter‐µm and number of cells per blastocyst; data expressed as mean ± SD) had no differences between groups (Chi‐Square test and Kruskal–Wallis test, *p* > 0.05) (*n* = 10 replicates).

Successful conjugation of pig and rabbit recombinant OVGP1 protein to NP, designated NPOv, was confirmed by immunoblot. When probed with anti‐OVGP1 (porcine) and penta‐histidine (porcine and rabbit) antibodies, bands were present with the expected 75 kDa molecular mass (**Figure** **1**A‐middle panels). The conjugation to NP was stable for at least 31 days (Figure 1A‐right panels) and NPOv was present uniformly on the ZP (Figure 1B) as the embryo developed from 2‐cells to blastocyst (Figure 1B) (tested with porcine eggs and embryos). NPOv distribution was assessed in eggs because of the homogeneity of their size (≈150 µm). The ZP area covered by NP was significantly lower when eggs were incubated with control NP compared to those incubated with NPOv after incubation for 0.5, 1, and 6 h (*p* < 0.05). The maximum area covered by NP (≈40%) was obtained after 1 h of incubation in the NPOv‐20 group (Figure 1B). These results provide evidence that NP specifically bound to the ZP when conjugated to OVGP1, although a low level of non‐specific binding of control NP was observed that was dependent on time of incubation.

To determine if NPOv bound to eggs had any effect on gamete function, we determined oxidative phosphorylation and glycolytic rates with OCR (oxygen consumption rate) and ECAR (extracellular acidification rate), respectively, after co‐incubation with NPOv using an extracellular flux analyzer. No differences were observed in OCR and ECAR parameters between NPOv‐eggs (10 and 20 groups) and the control group (without NPOv) (Figure 1C; Figure S1A, Supporting Information). NPOv incubation did not affect either sperm metabolism or motility (Figure S1B,C, Supporting Information). OVGP1 has been reported to be involved ZP hardening,^[^ ^38^ ^]^ a phenomenon which could impede sperm penetration during fertilization. To investigate this possibility, we assessed ZP hardening by a digestion assay and observed no effect of the presence of NPOv on digestion times (control = 45.76 ± 18.5 s; NPOv = 43.22 ± 15.77 s; *p* > 0.05) (Figure 1C). To further assess eggs competence, eggs bound to NPOv were fertilized in vitro (Figure 1D) and their developmental rates were compared to a control group. One that was not incubated with NPOv and another where NPOv were mechanically separated from eggs by gently pipetting until detachment of the NP to test any detrimental effect of NP separation. The presence of NPOv around the ZP at fertilization did not affect egg penetration, monospermy, and IVF efficiency (representing the final number of putative zygotes per 100 penetrated eggs) (*p* > 0.05) (Figure 1D upper graph). No differences were observed between experimental groups in terms of embryo development (cleavage and blastocyst rates) and quality (diameter and number of cells per blastocyst) (*p* > 0.05) (Figure 1D lower graphs).

### 2.2 Effect of NPOv on Reproductive Performance and Body Weight at Birth in Rabbits

**Figure 2:**
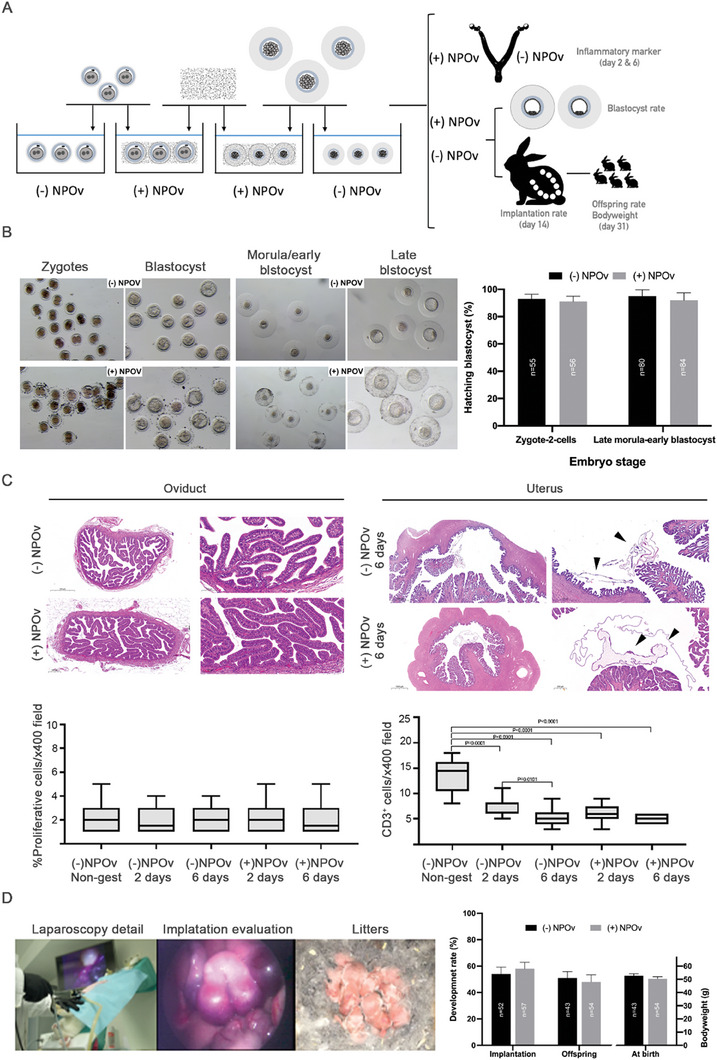
Reproductive performance and oviduct/uterine tissues are not affected after NPOv embryo transfer (rabbit model). A) Experimental design of NPOv application in the rabbit model. The schematic representation shows the incubation of embryos (zygote‐2 cells and late morula) with NPOv after in vivo collection and developed in vitro or transfer to host mothers. B) In vitro development of NPOv embryos shows a similar performance to their counterparts without NPOv. The first row of images displays the zygote‐2 cell stages and their in vitro development into blastocysts in the (‐)NPOv and (+)NPOv groups (Bar scale, 0.1 mm). The second row of images shows late‐morula embryos and their in vitro development into blastocysts in the (‐)NPOv and (+)NPOv groups (Bar scale, 0.1 mm). A total of 111 zygote‐2 cells and 109 morulas were used. The assessment of in vitro embryo development revealed similar results in both the zygote‐2 cell and morula stages between the two groups (‐NPOv and +NPOv) (*p* > 0.05). Data are expressed as mean ± SEM. C) The female reproductive tract is not affected after NPOv‐embryo transfer. A total of 120 (‐)NPOv and (+)NPOv zygote‐2 cells were transferred (d0) to 6 foster mothers, with the (+)NPOv zygote‐2 cells being placed in one oviduct and the (‐)NPOv zygotes in the other oviduct. After 2 and 6 days (d2 = 3 females and d6 = 3 females) post‐transfer, oviducts and uterine horns were collected for tissue evaluation. Representative histology micrographs with hematoxylin and eosin staining are shown. Box plots in the left graph indicate the rate (%) of proliferative cells (analyzed by ki67) not showing differences between groups (*p* > 0.05). Box plots in the right graph indicate the inflammatory reaction (CD3 positive cells, %) in oviduct and uterine tissues. The results indicate no differences between (‐)NPOv and (+)NPOv groups (*p* > 0.05). Black arrows in the histological images indicate embryonic tissue. D) In vivo development of NPOv‐embryos shows similar performance that their counterpart without NPOv. Images show the embryo transfer laparoscopic procedure, implantation evaluation, and litter. A total of 195 zygotes, 105 for (+)NPOv and 90 for (‐)NPOv, were transferred to ten recipients (five per each group). The transfers from the in vivo trial were conducted in two replicates. Regarding the in vivo results, consistent with what was observed in vitro, no differences were found in terms of implantation and live births (*p >* 0.05). Additionally, the body weight of the kits was similar between both groups (*p >* 0.05).

The OVGP1 rabbit protein was conjugated with NP and tested on two embryo stages (**Figure** **2**A): zygote‐2‐cells and late morula/early blastocyst. Following an additional 72 h for zygote‐2‐cells and 24 h for late morula/early blastocyst of in vitro culture, embryos exposed to NPOv exhibited a comparable developmental capacity to reach the hatching blastocyst stage when compared to the control group (92.0 ± 5.43% vs 91.0 ± 3.90%) (*p* > 0.05) (Figure 2B). These findings were similar to observations with pigs. Thus, early development was not perturbed by NPOv binding in rabbits and pigs. To further test the safety of NPOv, pronuclear embryos were collected 14 h post‐insemination, exposed to NPOv and transferred to foster mothers (Figure 2B, lower row). At day 2 and 6, respectively of the transfer, oviducts and uterine horns were collected for tissue evaluation. No histological abnormalities were found and cell proliferation, assessed by Ki‐67, was similar between groups (*p* > 0.05) (Figure 2C). The expression of CD3, a marker of inflammation, was not affected by the presence of NPOv (*p* > 0.05), although the female reproductive tract that did not receive embryos (non‐gest) showed a significant increase in CD3 positive cells compared with the rest of the groups (*p* ≤ 0.0001) (Figure 2C; Figure S2, Supporting Information). For the remaining foster mothers, there were no differences in terms of implantation (57/105 and 52/90 for (+)NPOv and (−)NPOv, respectively), offspring rate (54/105 and 43/90 for (+)NPOv and (−)NPOv, respectively), or body weight at birth between NPOv and control groups (*p* > 0.05) (Figure 2D).

### 2.3 Safe Magnetic Force‐Based Technique to Manipulate Eggs and Embryos

**Figure 3:**
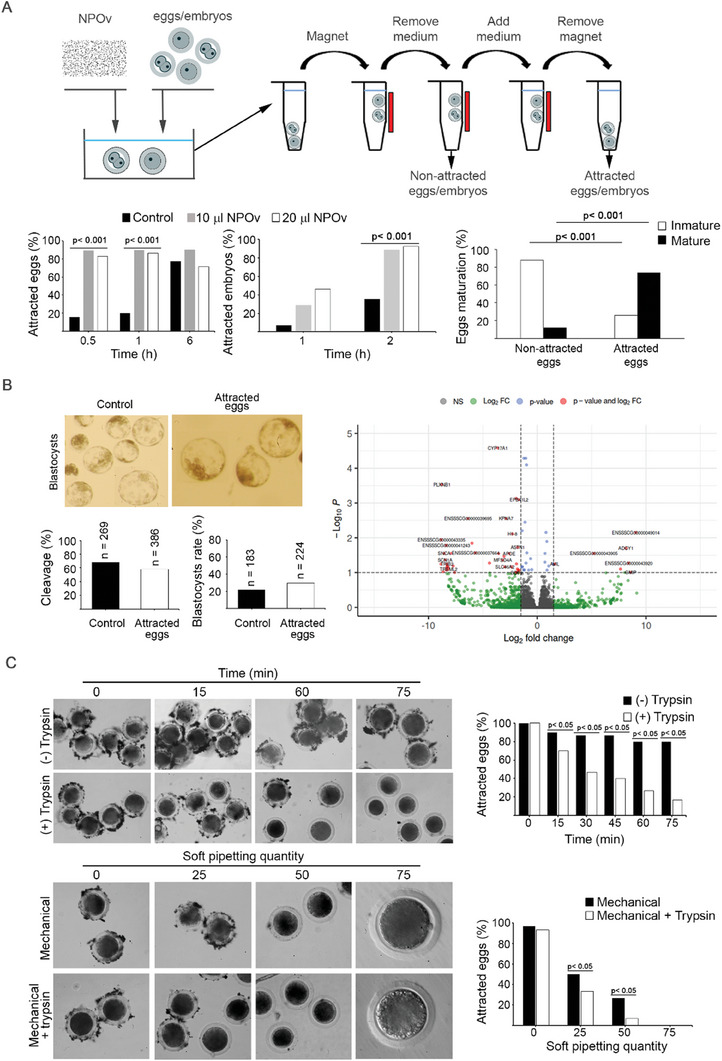
NPOv incubation with eggs/embryos results in an efficient (and reversible) union attracting them when subjected to a magnetic force without impairing further development. A) Eggs/embryos‐NPOv respond to a magnetic force. Eggs/embryos were co‐incubated with NPOv (upper panel). At each time point, eggs/embryos were placed in a 1.5 ml tube and subjected to a magnetic field (magnet, MagRack6, Cytiva). Thereafter, non‐attracted and attracted eggs/embryos were removed and counted. For the control group, eggs/embryos were incubated with NP in absence of Ov. The percentage of attracted eggs (left graph) was ≈80% for each time point evaluated (0.5, 1, and 6 h) (*n* = 5 replicates, a total of 322 eggs) (Chi‐Square test, *p* < 0.001). In the case of embryos, the greatest level of attraction was observed after 2 h of incubation (middle graph) (*n* = 4 replicates, a total of 120 embryos) (Chi‐Square test, *p* < 0.001). The non‐attracted and attracted eggs were fixed and stained (Hoechst 33342) to evaluate the level of maturation (right panel). More than 85% of non‐attracted corresponded to immature oocytes (*n* = 4 replicates, a total of 203 oocytes) (Chi‐Square test, *p* < 0.001). B) Embryo development of attracted eggs (NPOv‐eggs) has similar performance to eggs non‐subjected to a magnetic force. After IVF, zygotes were cultured in NCSU‐23 media and evaluated for cleavage (48 hpi) and blastocyst development (7 dpi). The gene expression of the embryos was analysed by RNAseq. To determine the differentially expressed genes (DEGs) the volcano plot shows Log_2_FC (*x*‐axis) against the *p*‐value (*y*‐axis) of significant DEGs (*p* ≤ 0.05 and Log_2_FC ≥1, red circles) (*n* = 4 replicates, each replicate was composed by a pool of ten blastocysts per group). C) Eggs‐NPOv union is reversible after chemical (trypsin) and/or mechanical (gentle pipetting) treatments. Trypsin presence removes the NPOv reducing the attraction when subjected to a magnetic field from 100% to <20% of eggs in 75 min (upper panel, right graph) (*n* = 3 replicates, a total of 120 eggs, Chi‐square test, *p* < 0.05). Mechanical treatment (combined or not with trypsin) reduced up to 0% of attracted eggs (lower panel, right graph) (*n* = 3 replicates, a total of 90 eggs, Chi‐square test, *p* < 0.05).

To take advantage of the intrinsic superparamagnetic parameters of the NP used in NPOv, we developed an assay to isolate eggs/embryos by magnetic force. For this purpose, we used a magnetic stand to evaluate the number of attracted and non‐attracted eggs/embryos under varying times and doses of NPOv. After ZP enclosed eggs were incubated with NPOv for 0.5, 1, and 6 h, ∼70% to 90% of the NPOv were attracted by magnetic force (**Figure** **3**A, left graph). The magnetic attraction of eggs incubated with NP alone increased with time (15.7% at 0.5 h; 20.0% at 1 h and 77.2% at 6 h) (Figure 3A, left graph). These results together with those shown in Figure 1 collectively suggest that at 30 min, 25% of the ZP was covered by NPOv and most of the NPOv eggs were attracted by a magnetic force. In the case of embryos, 2 h of NPOv‐incubation was necessary for most NPOv‐embryos (≈90%) to be attracted by magnets (Figure 3A, middle graph). We determined that some eggs were not attracted (≈20%) by the magnetic field. As the ZP is modified during egg maturation,^[^ ^39^ ^]^ we speculate that incompetent eggs would be less able to bind OVGP1. To test this hypothesis, we determined the maturation stage of attracted and non‐attracted eggs. Most of the eggs that were not attracted were immature (88% vs 12%), whereas most of those attracted were mature (74% vs 26%) (*p* < 0.001) (Figure 3A right graph). These data document that specific binding of NPOv to the surface of the ZP correlates with their maturation state and more mature eggs are attracted by magnetic forces.

To confirm the competence of NPOv coated eggs isolated by magnetic force, IVF was performed. Eggs were incubated for 30 min with NPOv, isolated by magnets and compared to non‐treated eggs. Development to cleavage (58.03% to 68.03%) and to blastocysts (21.86% to 29.46%) was similar in both groups (*p* > 0.05) (Figure 3B). Using RNA‐seq, we investigated any effect on the transcriptomes of blastocysts. A total of 15957 transcripts were detected by strand‐specific RNA‐seq of which 67 (14 up‐regulated and 53 down‐regulated) were differentially expressed (*p*  adj< 0.1). Only 46 genes (7 up‐regulated, 39 down‐regulated) were differentially expressed at log_2_FC (fold change) ≤1.5 (Figure 3B; Figure S3, Supporting Information). Additionally, oxidative stress was not altered in eggs (evaluated by intracellular ROS levels, *p* > 0.05) or embryos (evaluated by oxidative stress related‐genes, *p* > 0.05) when subjected to a magnetic field (Figure S4, Supporting Information). Based on these observations, binding magnetic NPOv to the ZP surrounding eggs and embryos for isolation, had no detrimental effect on development and a very minor effect on the transcriptome (<0.3%).

Although NPOv did not adversely impact fertilization and early development, we also investigated their removal from the ZP. We tested two protocols: chemical (trypsin) and/or mechanical (pipetting). Trypsin incubation for 75 min removed most NPOv from the ZP and reduced magnetic attraction from 100% to less than 20% of eggs (Figure 3C upper panels). Mechanical treatment (with or without trypsin) reduced magnetic attraction of NPOv coated eggs to 0% after 75 times of gentle pipetting (Figure 3C lower panels). There were no differences in zona matrix thickness between control and trypsin treated groups (19.05 ± 1.77 µm (n = 22) and 19.94 ± 1.54 µm, respectively (*n* = 23), *p* = 0.282).

### 2.4 Biophysical Characterization of Magnetic Force‐Based Manipulation Technique in ART

**Figure 4:**
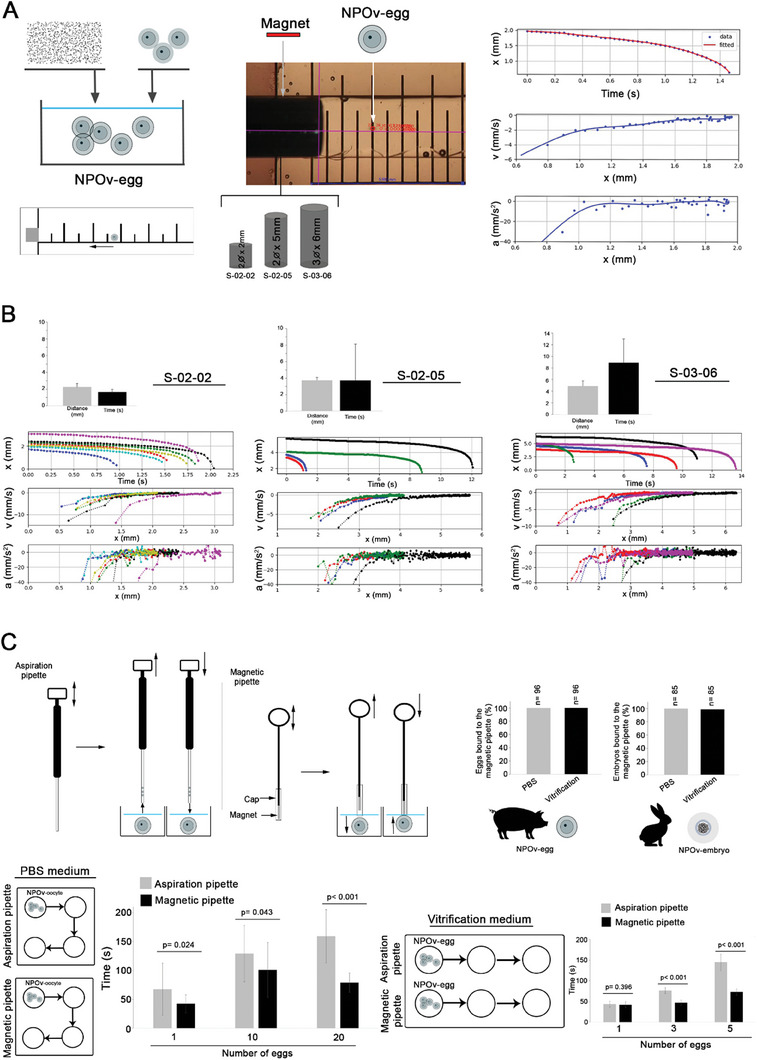
NPOv‐eggs/embryos responsiveness to a controlled magnetic force. A) Scheme of the experimental design where NPOv‐eggs were subjected to different magnetic fields [neodymium magnets: S‐02‐02 (2 mm ø × 2 mm height), S‐02‐05 (2 mm ø × 5 mm height), S‐03‐06 (3 mm ø × 6 mm height)] under a stereomicroscope to record the tracking of the movement (from the starting movement by the magnetic attraction to the attachment to the magnet). The time (s), distance (x, mm), velocity (v, mm ^−1^s) and acceleration (a, mm ^−1^s^2^) of the NPOv‐eggs were analyzed. An example of the position as a function of time for a particle initially located at ≈2 mm from a S‐02‐02 magnet is provided (dots in top‐right graph) and the position after smoothing by a polynomial fitting (solid line in the same graph). Velocity and acceleration of the particle, obtained by using central finite differences, as a function of its position are shown (dots in middle and bottom‐right graphs), the solid lines were calculated by analytical differentiation of the fitted x(t) curve. B) Kinematics of NPOv‐eggs under the action of the magnets presented in (A), where each column corresponds to the results obtained with different magnet: S‐02‐02 (graphs on the left column), S02‐05 (graphs on the middle column) and S03‐06 (graphs on the right column). Magnitudes of the effective range and the capture time (mean ± SD) for NPOv‐eggs (graphs on the top row), position as a function of time (graphs on the second row, from top to the bottom), velocity as a function of distance (graphs on the third row) and acceleration as a function of the distance (graphs on the bottom row) are shown for each magnet. C) NPOv‐eggs/embryos are effectively attracted by a magnetic pipette showing less time consuming than conventional pipettes. The aspiration pipette is the conventional method to move eggs/embryos in ART. A new system is provided using a magnetic pipette coupled to NPOv system to move eggs/embryos by a controlled magnetic force. Magnetic pipette was approached to eggs/embryos submerged in PBS or vitrification media and counted the number of attached structures. NPOv‐eggs/embryos respond in a very highly efficient manner (graphs on the right, upper row). The time consumed to move eggs media was reduced by the use of magnetic pipette in comparison with aspiration pipette in both, PBS (*n* = 4 replicates; 416 eggs) and vitrification media (*n* = 5 replicates; 45 eggs) (lower row).

After establishing an efficient method of egg/embryo isolation by NPOv and magnets, we focused on the dynamical behavior of NPOv‐egg under different magnetic forces (**Figure** **4**A,B; Figure S5, Supporting Information). NPOv‐eggs were exposed to magnetic fields previously characterized by a theoretical model (Figure S6, Supporting Information). To estimate the effective range of different magnets, NPOv‐eggs in PBS media were located on the axis of three magnets (designated S‐02‐02, S‐02‐05, S‐03‐06) at distances at which they were barely attracted (Figure 4A). The time needed for the NPOv‐eggs to reach the magnet was recorded. Figure 4B (first row of graphs) shows the effective range (in mm) and the capture time (in seconds) obtained for each magnet. Both parameters increased with the size of the magnet. The trajectory graphs (Figure 4B, second, third, and last rows) show the dynamics of NPOv‐eggs movement from their starting points to attachment on the magnets. NPOv‐eggs initially move at a slow speed and an almost null acceleration (i.e., the net force over the particle is nearly zero) independent of the magnet used. Therefore, the attracting magnetic force, which increases when approaching the magnet, is partially balanced with resistance forces. In this case, two different friction forces are postulated, a drag proportional to the speed that is due to movement in fluid, and a constant frictional force due to the contact between the surfaces on the petri dish and egg. The buoyant force exerted on the egg is less than gravity and the NPOv‐egg is touching the surface of the dish. When the distance between the magnet and the NPOv‐egg decreases, the magnetic force increases. The ferromagnetic response of the NPOv‐egg is linear for low magnetic force and becomes non‐linear for high magnetic force due to the magnetic force produced by the particle itself. Because of this non‐linear response, an abrupt change in the acceleration of the particle was observed. The distance at which occurs this drastic change in acceleration depends on the external magnetic field and the number of ferromagnetic NP on the surface of the ZP surrounding the egg.

Once fully evaluated, the ability of magnets to effectively isolate NPOv‐eggs may be of interest for ART. We suggest that magnetic‐pipettes would be superior to conventional aspiration for eggs/embryos isolation (Figure 4C). In this method, NPOv‐eggs/embryos were placed in media (PBS or vitrification media) and thereby, structures were manipulated based on the magnetic field with a high efficacy nearly to 100% in all the cases, independently of the structure (egg or embryo), species (rabbit or pig) or media (PBS or vitrification) (Figure 4C, upper row). Moreover, the time consumed to move eggs between wells, was reduced using the magnetic pipette in comparison with the aspiration pipette in both media used, PBS (P< 0.05) and vitrification (*p* < 0.001), except for one‐egg group when using the vitrification medium (*p* = 0.396) (Figure 4C, lower row).

## 3 Discussion

The ZP is a unique extracellular matrix that surrounds eggs and embryos^[^ ^40^ ^]^ during their transit through the oviduct to the uterus. Once there and dependent on a suitable hormonal milieu, embryos hatch from the zona matrix to implant in the endometrium.^[^ ^41^ ^]^ While the egg undergoes a dramatic cellular transformation to become an embryo, the ZP acts as a stable shield to protect it from the oviductal environment prior to implantation. This makes the ZP a suitable target for isolation of egg/embryos without impairing fertility or early development.

We describe a new technology to attach magnetized NP to the zona matrix for isolation of eggs and embryos. OVGP1 is the most abundant protein in the oviduct and uterus that binds to the ZP in a wide range of mammals.^[^ ^42^ ^]^ The N‐terminal domain is highly conserved among species and is responsible for binding to the ZP. The C‐terminal domain varies among mammals and dictates its order‐specific function including the ability to penetrate through zona matrix.^[^ ^37^ ^]^ We used recombinant OVGP1 proteins (pOVGP1t, 481 aa; rOVGP1, 475 aa) attached to NP that prevents internationalization. Our studies demonstrate that short‐term incubation of eggs/embryos with NPOv is sufficient for targeted binding to the outer aspect of the zona matrix. The attachment of NPOv or its presence in media was neither detrimental to fertilization nor embryonic development. Even after removal of NPOv, eggs could be efficiently fertilized and developed in vitro to blastocysts. Co‐incubation of NP with eggs and embryos had no harmful effects.^[^ ^24^, ^25^, ^43^ ^]^ NPOv‐embryos transferred in vivo into rabbit uteri had no adverse morphological effect on the endometrium at implantation^[^ ^44^, ^45^ ^]^ and there was no effect on the number of offspring or body weight at birth.

Despite advances, developmental rates of in vitro produced embryos are suboptimal^[^ ^46^, ^47^ ^]^ and it is important to assess microfluidics and micro‐nanoscale devices^[^ ^6^ ^]^ that approximate in vivo conditions. These technologies need to precisely guide eggs/embryos through pre‐designed medical equipment organized in 2‐ and 3‐dimensions^[^ ^48^ ^]^ and can be applied to ART. Magnetic NP permit manipulation of eggs/embryos without physical contact. With a brief co‐incubation of eggs with NPOv, most in vitro matured eggs can be retrieved by a magnetic field. Most eggs recovered were competent to be fertilized in vitro while those that failed to be attracted by the magnet were immature which is a further advantage of this technology. Selecting cells with magnets can have adverse effects depending on the magnetic field intensity^[^ ^49^, ^50^ ^]^ but with relatively weak magnets, we have not observed any adverse effects on eggs and embryos. Eggs rescued by magnetic force and fertilized in vitro have normal development and their transcriptomes are not significantly different from controls. We further demonstrated that with simple pipetting or very light trypsin treatment, the eggs lose their ability to be attracted to the magnetic field. Thus, the magnetic‐attraction property acquired by the presence of NPOv in the outer part of the ZP is reversible.

The trajectory and speed of egg movement were characterized to explore the automatization of the movement of reproductive cells by magnetic force. Following the protocol described here, all eggs behaved similarly and with similar kinetics, so magnetic manipulation of eggs could be a crucial tool to guide cells toward pre‐designed organizations in 3D cell cultures (i.e., oviduct‐on‐a‐chip). Moreover, cell manipulation using magnetic labels offers high purity, selectivity, and recovery rates for cell separation.^[^ ^48^ ^]^ Processes including in vitro maturation, in vitro fertilization, embryonic culture, and development or vitrification require the manipulation of eggs and embryos to provide required media and reagents. Here we provide evidence in proof‐of‐concept experiments that eggs can be easily and quickly manipulated by magnetic pipettes using different conditions to avoid manipulation by aspiration. This may be an advantage for the manipulation of groups of eggs and embryos recovered from a single female for processing. However, additional studies are necessary to establish the safety of the intervention on human subjects and other animal species.

In summary, we have determined that ferric NP attached to OVGP1 (NPOv) and bound to the ZP can manipulate eggs and embryos. Using NPOv, competent matured eggs were retained by an external magnetic field for use in ART while non‐competent eggs were discarded. We have demonstrated that NPOv‐eggs/embryos can be moved in a desired direction which augurs well for use in state‐of‐art microfluidic technologies (i.e., a complete on‐chip IVF platform). In addition, we have demonstrated that eggs and embryos can be manipulated using magnetic pipettes to avoid mechanical aspiration. We have thus demonstrated a robust, non‐toxic technique that can potentially be used for gamete selection, embryo culture, and represents a new paradigm that facilitates entrance into a new era of ART.

## 4 Experimental Section

### Reagents

Unless mentioned, reagents used were provided by Sigma‐Aldrich (Madrid, Spain).

### OVGP1‐Nanoparticles Conjugation

Recombinant truncated porcine and rabbit OVGP1 plasmid expression, protein production, and purification were previously described.^[^ ^37^ ^]^ Carboxyl‐Modified Paramagnetic particles (‐COOH) (Estapor) (NP) with a diameter of 0.365 µm and a concentration of 1 mg ml^−1^ were used in this study. A magnetic rack (Cytiva Life Sciences MagRack6, Fisher Scientific) was used to handle NP. 10 µl of NP previously washed twice in 500 µl of milli‐Q water after a vigorous vortex agitation were used for conjugation. First, the NP surface was activated by re‐suspending in 240 µl of activation buffer (sodium phosphate 100 mm, pH 6.2), with 30 µl of conjugation buffer 1‐(3‐dimethylaminopropyl)−3‐ethylcarbodiimide HCl or EDC (ProteoChem, Hurricane, UT, USA) (50 mg ml^−1^ diluted in water) and 30 µl of conjugation buffer Sulfo NHS (50 mg ml^−1^ diluted in water) (ProteoChem, Hurricane, UT, USA). Afterward, gentle agitation for 20 min at room temperature (RT) was performed. Subsequently, NP were washed twice in 500 µl of coupling buffer (sodium bicarbonate 0.1 m, pH 8). Then, NP were incubated with 6 µg of pOVGP1t in 300 µl of coupling buffer (sodium bicarbonate 0.1 m, pH 8) at RT for 2 h. Finally, NP were washed twice in sodium phosphate buffer 20 mm and kept in this media at 4 °C until use in a final volume of 400 µl. An electrophoresis and immunoblot with a polyclonal anti‐OVPG1 (Abcam, Cambridge, United Kingdom) antibody were performed to test the conjugation. Image analyzer ImageQuant LAS 500 was used to detect the protein.

### Porcine Gamete Collection, In Vitro Fertilization (IVF), and Embryo Production

Cumulus‐oocyte complexes (COCs) were obtained from ovaries (prepuberal females) collected at a local slaughterhouse and processed.^[^ ^51^ ^]^ Briefly, COCs were collected by aspiration from antral follicles (3–6 mm diameter) and washed in Dulbecco's PBS with 1 mg ml^−1^ polyvinyl alcohol (DPBS‐PVA). The COCs (50‐55 per well) were incubated in 500 µl of NCSU‐37a medium previously balanced for 3 h at 38.5 °C/5% CO_2_ for 20–22 h in a Nunc four‐well dish. Subsequently, the COCs were transferred to 500 µl of NCSU‐37b medium free of eCG, hCG, and dibutyryl AMPc, where they were incubated for another 20–22 h under the same conditions.^[^ ^52^ ^]^ Porcine IVF was performed with sperm (sperm‐rich fraction from proven fertile boars) previously selected by discontinuous Percoll gradient and the concentration was adjusted to 1.5 × 10^6^ sperm/ml.^[^ ^53^ ^]^ For porcine embryo culture, putative zygotes were transferred to embryo culture medium NCSU‐23a for 24 h at 38.5 °C, 5% CO_2,_ and 7% O_2_. At 48 h post‐insemination (hpi), the cleavage rate was evaluated, and 2–4 cell embryos were transferred to embryo culture medium NCSU‐23 until day 7 post‐insemination (dpi) when blastocyst formation rate was evaluated.^[^ ^52^ ^]^

### Collection of Rabbit Embryos and In Vitro Development

Fifteen nulliparous New Zealand White females underwent superstimulation using a combination of FSH (Corifollitropin alfa, 3 µg, Elonva, Merck Sharp & Dohme S.A.) and hCG (7.5 UI).^[^ ^54^ ^]^ After 72 h of superstimulation, the females were inseminated with pooled semen from New Zealand bucks of proven fertility. Ovulation was induced with 1 µg buserelin acetate (Suprefact; Hoechst Marion Roussel, S.A., Madrid, Spain). Females were euthanized in two groups, at 22 h (*n* = 9) and 72 h (*n* = 6) after artificial insemination, and the reproductive tract were immediately removed. Zygotes and two‐cells, and late morulae and early blastocyst embryos were recovered by washing each uterine horn with 10 ml of DPBS containing 0.2% (wt/vol) bovine serum albumin (BSA). The collected embryos were counted and evaluated according to IETS criteria. At 22 h, only zygotes (two corpuscles and two pronuclei) and 2‐cell embryos were categorized as suitable embryos, while at 72 h, embryos at the late morula and early blastocyst stages, exhibiting a homogeneous cell mass, a spherical mucin layer and ZP, were categorized as suitable embryos.

### NPOv and Egg/Embryos Incubation and Attachment Evaluation by Microscope

Eggs or embryos were co‐incubated with NPOv (10 or 20 µl) in 500 µl of DPBS‐BSA in a Nunc 4‐well dish for at least 20 min at 38.5 °C (porcine) or at 22–25 °C (rabbit). The percentage of porcine egg surface cover by NPOv was measured with a computer‐assisted image analyzing system (Q5501W, Leica Microsystem Imaging Solutions Ltd, Cambridge, United Kingdom) using Leica Qwin Pro, Version 2.2 software. This system acquires high‐definition digital images of the sample. The area corresponding to the NPOv and complete egg were selected from automatic threshold of grey levels of the NP and the egg, respectively. For scanning electron microscope (SEM) groups of 10–20 in vitro matured and denuded porcine eggs were fixed in 2% of glutaraldehyde at 4 °C for 2 h followed by three washed in DPBS. The evaluation was carried out using the scanning electron microscope ApreoS (Thermo Fisher Scientific).

### Metabolic Activity Analysis in Gametes

Metabolic measurements were assessed after gametes‐NPOv co‐incubation. Seahorse XFe extracellular flux analyzer (Agilent Technologies, Inc., CA, EE. UU) with 96‐well cell culture plate was used to measure metabolic status by real‐time oxygen consumption rate (OCR, pmol of min^−1^) and extracellular acidification rate (ECAR, milli‐pH min‐1). The seahorse assay plate was equilibrated and calibrated with sterile water (sensor cartridge placed on top) overnight at 37 °C in absence of CO_2_. The Real‐Time ATP Rate Assay kit was employed. After calibration, the 96‐well plate was loaded with eggs (20 eggs per well) or sperm (1 × 10^6^ sperm/well) in a final volume of 50 µl per well. For background corrections, four wells were left without cells. After 20–30 min of reading, a report was generated, and the data were collected and analyzed.

### Assessment of ZP Digestion

Matured eggs without cumulus cells were washed twice in PBS before being placed in 50 µl droplets of 0.5% pronase (wt/v in PBS). The dissolution of the ZP was continuously observed under a stereomicroscope. The time required for complete dissolution of the ZP was recorded.

### Evaluation of Porcine Oocyte Maturation, IVF, and Embryo Development

For the evaluation of oocyte maturation or IVF output, the cells were fixed for 15 min in 10% glutaraldehyde in PBS, stained for 15 min with 1% Hoechst 33342 in PBS, washed in PBS and mounted on glass slides for evaluation by epifluorescence microscopy. An oocyte was considered mature (egg) when the nucleus was in metaphase II stage and the first polar body was extruded. In the case of IVF, three parameters were calculated: penetration rate (%) (percentage of eggs with one or more male pronuclei of total eggs); monospermy rate (%) (percentage of penetrated eggs with only one male pronuclei), and efficiency rate (%) (percentage of eggs that were penetrated and monospermic from number of eggs inseminated).

For embryo culture evaluation the cleavage rate (at 48 hpi) and the blastocyst rate (on day 7 dpi) were evaluated. On day 7 dpi blastocysts were photographed, and image analyses were performed by ImageJ software for diameter analysis. Furthermore, blastocysts were fixed for 15 min in 10% glutaraldehyde in PBS, stained for 15 min with 1% Hoechst 33342 in PBS, washed in PBS, and mounted on glass slides for later evaluation of number of cells by epifluorescence microscopy.

### Evaluation of Rabbit In Vitro Embryo Development

Zygotes‐2 cell embryos and late morulae‐early blastocyst embryos were cultured in vitro for 72 and 24 h, respectively. The culture was conducted in four‐well multidish plates with 500 µl of TCM199 supplemented with 10% fetal bovine serum. The incubation temperature was maintained at 38.5 °C in a 5% CO_2_ air environment. Following the in vitro culture, the embryos were morphologically evaluated for their developmental progression up to the hatching blastocyst stage using a stereomicroscope.

### Implantation Rate, Offspring Rate, and Body Weight at Birth in the Rabbit Model

Zygotes and 2‐cell embryos (NPOv and control) were transferred according to the previously described method.^[^ ^55^ ^]^ Briefly, ovulation was induced in 16 receptive females (determined by vulva color) through the administration of 1 µg i.m. of buserelin acetate (Hoescht, Marion Roussel, Madrid, Spain). On the day of the embryo transfer, foster mothers were anesthetized by an i.m. injection of 4 mg kg^−1^ of xylazine (Bayer AG, Leverkusen, Germany), followed 5–10 min later by an intravenous injection into the marginal ear vein of 0.4 ml kg^−1^ of ketamine hydrochloride (Imalgene 500, Merial SA, Lyon, France). During laparoscopy, 3 mg kg^−1^ of morphine hydrochloride (Morfina, B. Braun, Barcelona, Spain) was administered intramuscularly. The embryo transfer was performed by laparoscopy, introducing the zygotes‐2 cells embryos into the oviducts (≈10 per oviduct). After the transfer, females were treated with antibiotics (4 mg kg^−1^ of gentamicin every 24 h for 3 days, 10% Ganadexil, Invesa, Barcelona, Spain) and analgesics (0.03 mg kg^−1^ of buprenorphine hydrochloride, [Buprex, Esteve, Barcelona, Spain] every 12 h for 3 days and 0.2 mg kg^−1^ of meloxicam [Metacam 5 mg mL^−1^, Norvet, Barcelona, Spain] every 24 h for 3 days). For the evaluation of the inflammatory response to NP in the oviduct and uterus (see below), each foster mother (*n* = 6) received NPOv zygotes‐2 cell embryos in one oviduct and control zygotes‐2 cell embryos in the other. The transfers to either the right or left oviduct were randomized. In the remaining recipient females (*n* = 10, with 105 and 90 embryos transferred for (+)NPOv and (‐)NPOv, respectively), the survival rate was assessed using laparoscopy, following the aforementioned procedure. The assessment included recording the implantation rate (number of implanted embryos on day 14 out of the total embryos transferred) and the birth rate (offspring born/total embryos transferred). Additionally, the body weight of the offspring was measured at birth.

### Inflammatory Response in the Oviduct and Uterine of Rabbits

Two and six days following the transfer of zygotes‐2 cell embryos, six foster mothers were euthanized, and reproductive tracts were collected from each experimental group (NPOv and control). Oviducts and uterine horns were fixed in 10% buffered formalin for 24 h, processed, and embedded in paraffin. Sections (3 µm) from samples were then stained with a hematoxylin and eosin (H&E) stain for conventional histopathological evaluation and to determine embryo implantation. To determine changes of proliferative rate of epithelial cells from oviducts, an indirect polymer‐HRP labelled immunohistochemical procedure was performed to study the expression of Ki‐67 (Agilent, Dako) protein. Positive immunoreaction was evidenced by a dark‐brown precipitated with a nuclear pattern. Proliferative rate was estimated by the median percentage of positive epithelial cells in ten high‐power fields (HPF, X400). Additionally, an anti‐TCD3 (Agilent, Dako) IHC was also incubated with uterine tissues to study the degree of T‐cell inflammatory infiltrate (with a dark‐brown pericellular membrane pattern), determining the median of positive cells in 10 HPF. Immunohistological examinations were performed by using a direct‐light conventional microscope (Zeiss Axio Scope A10, Carl Zeiss, Jena, Germany). Representative images were obtained by a high‐resolution digital camera (Zeiss AxioCam 506, Zeiss) using Zeiss ZEN (vs 3.0) software.

### Analysis of NPOv‐Egg/Embryo Attraction by a Magnetic Field

After incubation, cellular structures (NPOv‐eggs or embryos) were re‐suspended in PBS and transferred to a 1.5 ml centrifuge tube. The tube was then placed in a magnetic rack (Cytiva Life Sciences MagRack6, Fisher Scientific) and the NPOv‐eggs/embryos in suspension were pulled to the side of the tube by the magnet. The supernatant was then removed (carrying the non‐attracted structures) and new medium was added in the tube. Finally, the magnet was removed, containing the tube the attracted structures.

### Reversibility Assessment of NPOv to Porcine Eggs

The reversibility of the NPOv bound to the ZP of the eggs was evaluated by chemical (trypsin) and/or mechanical (gentle pipetting). For chemical treatment, NPOv‐eggs were incubated with trypsin (0.5%) or not (control group). The attraction of the NPOv‐eggs (%) was assessed using a magnetic rack (Cytiva Life Sciences MagRack6, Fisher Scientific) at 0, 15, 30, 45, 60, and 75 min of incubation in both groups. Moreover, photographs of NPOv‐eggs from both groups were taken at the end of the evaluation period to determine ZP thickness using ImageJ software. For mechanical treatment, NPOv‐eggs were subjected to a gentle pipetting (0, 25, 50, or 75 movements) with or without trypsin (0.5%). The attraction of the NPOv‐eggs (%) was evaluated using a magnetic rack for mechanical and mechanical+trypsin groups.

### Embryo RNA‐Seq and Analysis

Blastocysts on day 6 dpi were stored in RNAlater at −80 °C for gene evaluation by RNA‐seq. For this purpose, RNA was extracted using the QIAamp RNA miRNeasy Micro Kit (QIAcube/QIAGEN) according to the manufacturer's instructions and the quality control (RNA Integrity Number) was assessed with a Bioanalyser System 2100 (Agilent Technologies, CA, USA) using an RNA 6000 Nano Kit. TruSeq Stranded Total RNA kit (Illumina, CA, USA) was used to make libraries from 150–300 ng total RNA input according to the manufacturer's protocol. First, the ribosomal RNA (rRNA) was removed, and the depleted RNA fragmented. Following this step, first and second strand cDNA synthesis were performed. The 3 ‘ends were adenylated and the index adapters ligated which prepared libraries for hybridization onto a flow cell. Finally, libraries were enriched by PCR, concentration and quality determined, normalized and pooled. Total RNA pooled libraries were sequenced using paired‐end 75 base sequencing chemistry on NextSeq 500 (Illumina, San Diego, CA, USA) following the manufacturer's protocol with sequencing depths ranged from 80 to 90 million paired‐end reads.

For RNA‐seq data analysis, the 15957 genes obtained after filtering the data (genes that did not have reads in any of the samples were eliminated, and then genes that did not have a minimum of 10 reads in at least 3 samples were eliminated). To determine differentially expressed genes (DE), a cutoff of 0.1 was established for padj and a log_2_FC >1.5 in absolute value (log_2_FC >1.5 or <−1.5). For this, the EnhancedVolcano (Blighe K, Rana S, Lewis M. EnhancedVolcano: Publication‐ready volcano plots with enhanced colouring and labeling. R package version 1.13.2. 2022) R package was used. They have been made with the DE genes following the same criteria established for the volcano plot, using for this the pheatmap (Kolde R. Pheatmap: Pretty Heatmaps. R package. Version 1.0.12. 2019. https://cran.r‐project.org/package=pheatmap (accessed April 20, 2022) function from R [this would be heatmapDEgenes.png] or the heatmap.2 function from the gplots (Warnes G, Bolker B, Bonebakker L, Gentleman R, Huber W, Liaw A, et al. Venables gplots: Various R Programming Tools for Plotting data. R package. Version 3.1.1. 2020. https://cran.r‐project.org/package=gplots (accessed April 20, 2022) package of R [this would be heatmapDEgenes2.png]’’.

### Measurement of Intracellular ROS (Reactive Oxygen Species) Levels

The oxidative stress of porcine eggs was analyzed by intracellular ROS (reactive oxygen species) formation using a DCFDA/H2DCFDA‐Cellular ROS Assay Kit (ab113851, Abcam, USA) and performed according to the manufacturer's instructions. Briefly, denuded eggs were incubated with 20 µm DCFDA in PBS containing 0.1% BSA for 45 min at 38.5 °C in the dark. After incubation, eggs were washed twice in PBS and then placed on glass slides. The fluorescent signal (in individual eggs) was evaluated immediately by a fluorescence microscope (Leica DMC6200). The fluorescent intensity was analysed using Leica Application Suite X software after normalization through subtraction of the background intensity to that of control eggs.

### Analysis of NPOv‐Eggs Tracking When Subjected to a Magnetic Field

NPOv‐egg was under the effect of an external magnetic field produced by commercially available neodymium magnets (Supermagnete, Gottmadingen, Germany), that were previously characterized by measuring the axial component of the magnetic field (13610.93 Teslameter and 13610.01 Axial Hall Probe, PHYWE Systeme GmbH & Co. KG, Göttingen, Germany) (Figure S6, Supporting Information). The dynamics of the NPOv‐eggs were recorded under a stereomicroscope coupled to a camera and later digitalized (Tracker 6.0.8 software, https://physlets.org/tracker/). The temporal evolution of the position, as well as the velocity and the acceleration, were plotted as a function of the position, where velocity and acceleration were calculated by using finite differences.

### NPOv‐Egg/Embryos Movement through a Magnetic Pipette

The efficiency of porcine eggs and rabbit embryos displacement between wells using magnetic fields was evaluated in PBS and vitrification media (Kitazato, BioPharma, Shizuoka, Japan). First, NPOv‐eggs/embryos were placed in four‐well plates and the number of attached structures was counted in both media when the magnetic field was immersed on them. Moreover, the time spent to move porcine NPOv‐eggs (in groups of 1, 10, and 20 eggs) from well 1 to well 4 (in total three eggs passages) using magnetic versus aspiration pipettes (The Stripper, CooperSurgical) was evaluated. Additionally, NPOv‐eggs were subjected to vitrification (Kitazato, BioPharma, Shizuoka, Japan) in groups of 1, 3, and 5 eggs to compare the time with each pipette. The vitrification process ends with the attachment of NPOv‐eggs to the cap of the magnetic pipette in the last step or positioning the NPOv‐eggs in the Cryotop (Kitazato, BioPharma, Shizuoka, Japan) using the aspiration pipette.

### Statistical Analysis

Statistical analysis for sperm parameters was performed using SAS University edition (SAS, 2016) software. All the motion parameters were compared with the mixed model of SAS. For other results, statistical analysis was performed using IBM SPSS v.23 (SPSS Inc. Chicago, IL, USA). Pearson Chi‐squared test was used to analyze percentage data (penetration, monospermy, efficiency, cleavage, and blastocyst rate). For embryo diameter and embryo cell number, Kruskal Wallis was used to determine statistics after a normality test by Kolmogorov‐Smirnov. For Seahorse results a Shapiro‐Wilks test was made for normality assessment, since both parameters showed normal distribution an ANOVA test was used. For ZP digestion time data a Kolmogorov‐Smirnov test was carried out for normality evaluation and U‐Mann Whitney test for groups comparison. For ROS data the Shapiro‐Wilks test was carried out for normality evaluation and Kruskal–Wallis test for groups comparison.

A generalized linear model (GLM) including the rabbit embryo [(+)NPOv and (‐)NPOv] as fixed effects was used. The error was designated as having a binomial distribution using probit link function. Binomial data for in vitro development, implantation rate, and offspring rate at birth were assigned as 1 if positive development had been achieved or a 0 if it had not. Also, a GLM was fitted for body weight analysis including the experimental group as a fixed effect and common litter as a random effect. Differences were considered statistically significant at *p* < 0.05.

### Ethics Approval Statement

The procedures involving porcine species were approved by the Ethical Committee of the University of Murcia on 1 June 2020 (reference project PID2019‐106380RB‐I00 and ethical committee reference 567/2019). The animal study protocol involving rabbits was reviewed and approved by the “Universitat Politècnica de València” Ethical Committee prior to initiation of the study (research code: 2018/VSC/PEA/0116). Animal experiments were conducted in an accredited animal care facility (code: ES462500001091). All experiments were performed in accordance with relevant guidelines and regulations set forth by Directive 2010/63/EU EEC.

## Conflict of Interest

M.J.M and F.A.G.V. are inventors on patent applications/patents related to the technology of particles attachment to the zona pellucida. The other authors of this study declare that they do not have anything to disclose regarding funding or conflict of interest with respect to this manuscript.

## Supporting information

Supporting Information

Supplemental Video 1

## Data Availability

The data that support the findings of this study are available in the supplementary material of this article.
