# Supplementary material for: Magnetic‐Assisted Control of Eggs and Embryos via Zona Pellucida‐Linked Nanoparticles
Source: Adv Sci (Weinh). 2024 Mar 6;11(18):2306901. doi: 10.1002/advs.202306901 (PMC11095233; doi:10.1002/advs.202306901)
Supplement: Supplementary file 1 — Supporting Information [file ADVS-11-2306901-s001.pdf]

ADVANCED SCIENCE
Open Access

Supporting Information

for *Adv. Sci.*, DOI 10.1002/advs.202306901

Magnetic-Assisted Control of Eggs and Embryos via Zona Pellucida-Linked Nanoparticles

Francisco Alberto García-Vázquez, Gabriela Garrappa, Chiara Luongo, Julieta Gabriela Hamze, María Caballero, Francisco Marco-Jiménez, José Salvador Vicente Antón, Gregorio J. Molina-Cuberos and María Jiménez-Movilla**

Supplementary Materials for

MAGNETIC-ASSISTED CONTROL OF EGGS AND EMBRYOS VIA ZONA PELLUCIDA-LINKED NANOPARTICLES

García-Vázquez FA*[1,2], Garrappa G[1,3], Luongo C[1], Hamze JG[3], Caballero M[1, 3], Marco-Jiménez F[4], Vicente JS[4], Molina-Cuberos G[5], Jiménez-Movilla M[2, 3]*

The PDF file includes:

Fig. S1. Gametes functionalities are not compromised by NPOv presence.

Fig. S2. Influence of NPOv-embryo transfer in the rabbit host uterus.

Fig. S3. Hierarchical clustering of samples and heatmap of differentially expressed genes between attracted and non-attracted oocytes (control).

Fig. S4. NPOv and magnetic force do not affect oxidative stress in eggs/embryos.

Fig. S5. Video showing an NPOv-egg (red dots) under the influence of a magnetic force (magnet in the left part of the image).

Fig. S6. Characterization of the magnets.

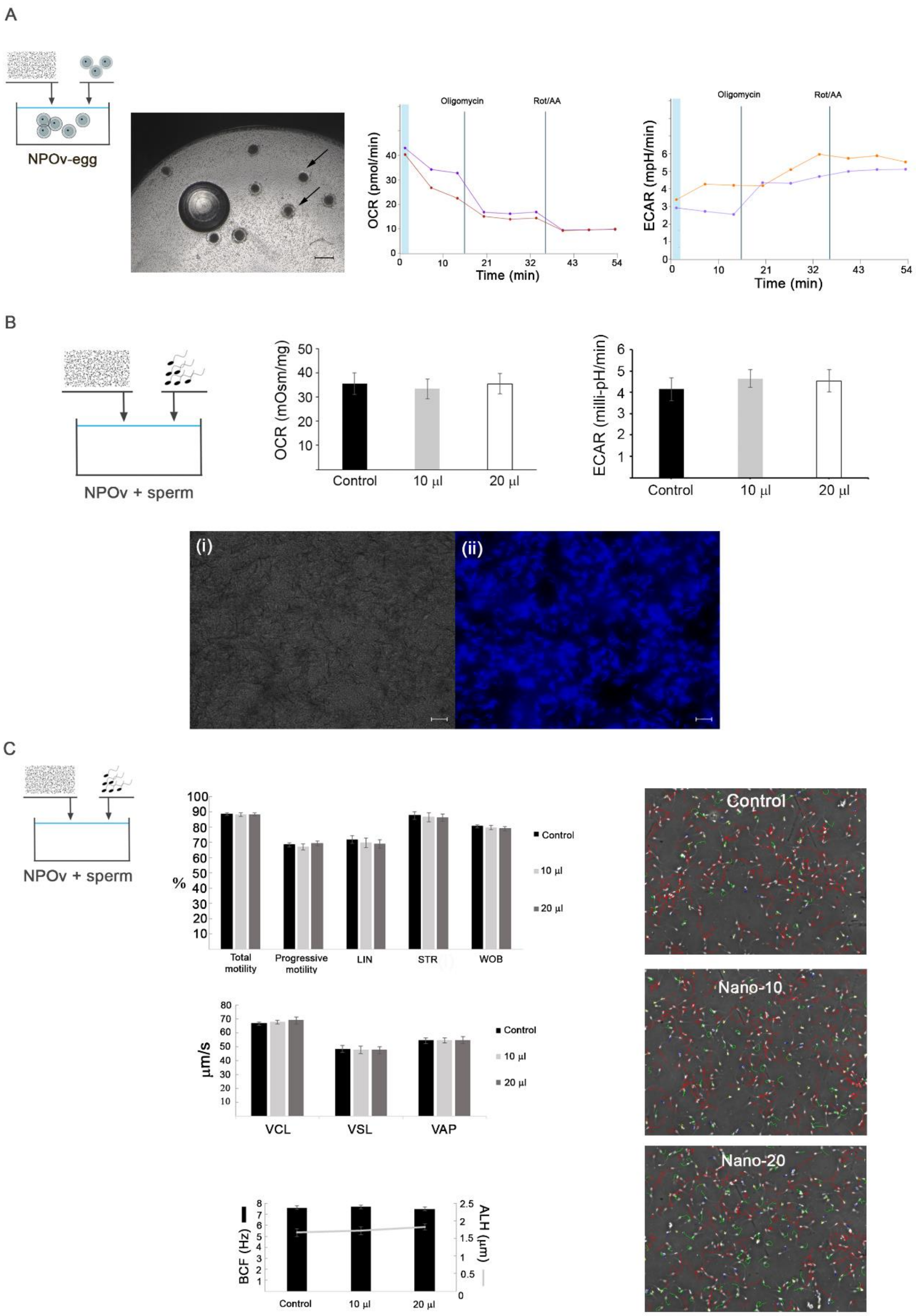

Fig. S1. Gametes functionalities are not compromised by NPOv presence. (A) Eggs (left image, black arrows indicate two of the eggs included in a replicate, Bar scale 200 µm) attachment during metabolic measurement in the XF Analyzer. An example of oxygen consumption rate (OCR) and extracellular acidification rate (ECAR) of eggs following oligomycin and rotenone

injections by the XF Analyzer. **(B)** Sperm metabolism (oxygen consumption rate-ORC, pmol/min and extracellular acidification rate-ECAR, milli-pH/min) are not influenced by NPOv presence (P> 0.05). Metabolic measurements were performed with Seahorse XFe96 (Agilent Seahorse analyzer, Agilent Technologies). Data are presented as mean ± SEM (n=8 replicates). Sperm cells ((i) brightfield; (ii) fluorescence-Hoechst staining, Bar scale 20 µm) attachment during metabolic measurement in the XF Analyzer. **(C)** Kinetics of sperm after co-incubation with NPOv. Sperm were incubated in TALP medium at 38.5 ºC with 5% CO_2 for 60 min (analyzed at 0, 30, and 60 min). Sperm in the control group were incubated without NPOv. Motion parameters were evaluated by a Computer Assisted Semen Analysis (CASA) (ISAS® software, PROiSER R+D S.L., Valencia, Spain) coupled to a phase-contrast microscope (negative-pH 10x objective; Leica DMR, Wetzlar, Germany) and a digital camera (Basler Vision, Ahrensburg, Germany). Assessed parameters were total motility (MOT) and progressive motility (PMOT), mean velocity-VAP (µm/s), linear velocity-VSL (µm/s), curvilinear velocity-VCL (µm/s), mean lateral head amplitude-ALH (µm), frequency of head displacement-BCF (Hz), straightness coefficient-STR (%), linearity coefficient-LIN (%). At least 3 different fields were analyzed per sample. Motility determinations were made at 25 frames per second for one second (25 images). Data are presented as mean ± SEM (n=5) (P> 0.05) (Proc Mixed procedure). Right images show sperm trajectories sequences (2 s) obtained by CASA system (ISAS®, PROISER R + D S.L., Valencia, Spain) (50 frames/s). Red trajectories indicate fast sperm (VAP > 45 µm/s), green medium speed (VAP >25 and ≤45 µm/s), blue low sperm speed (VAP ≤25 and >10 µm/s), and yellow static sperm (≤10 µm/s).

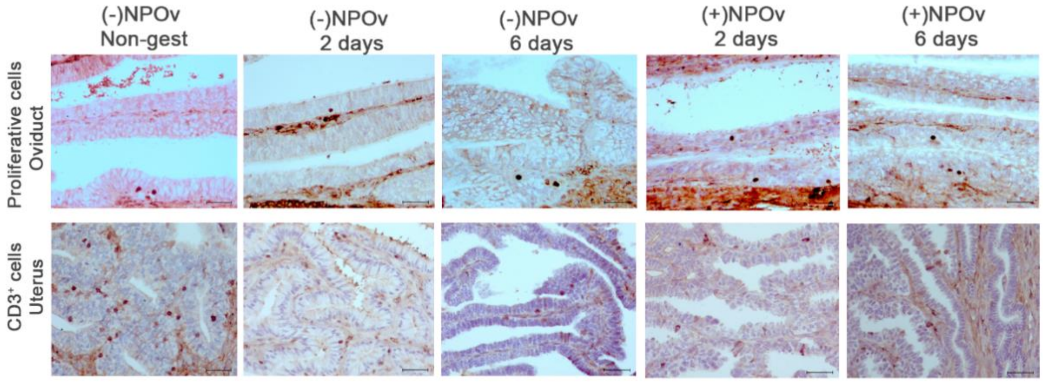

Fig. S2. Influence of NPOv-embryo transfer in the rabbit host uterus. After embryo transfer to the oviduct, the female genital tracts were collected (d2 and d6 after transferring) and fixed (10% buffered formalin) for 24 h. (-)NPOv indicates embryos not incubated with NPOv; (+)NPOv indicates embryos incubated with NPOv. Representative immunostaining images of Ki-67 (first row) and CD3 (second row) in rabbit oviduct and uterus respectively.

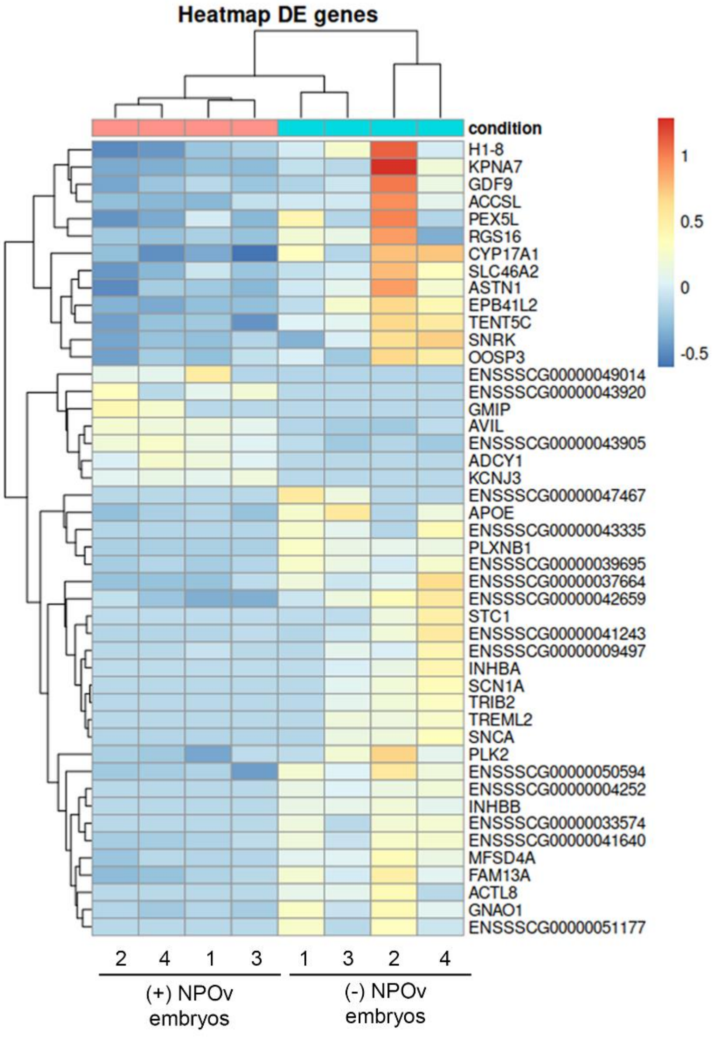

Fig. S3. Hierarchical clustering of samples and heatmap of differentially expressed genes between attracted and non-attracted oocytes (control). Normalized expression was plotted on a high-to-low scales (red-white-blue).

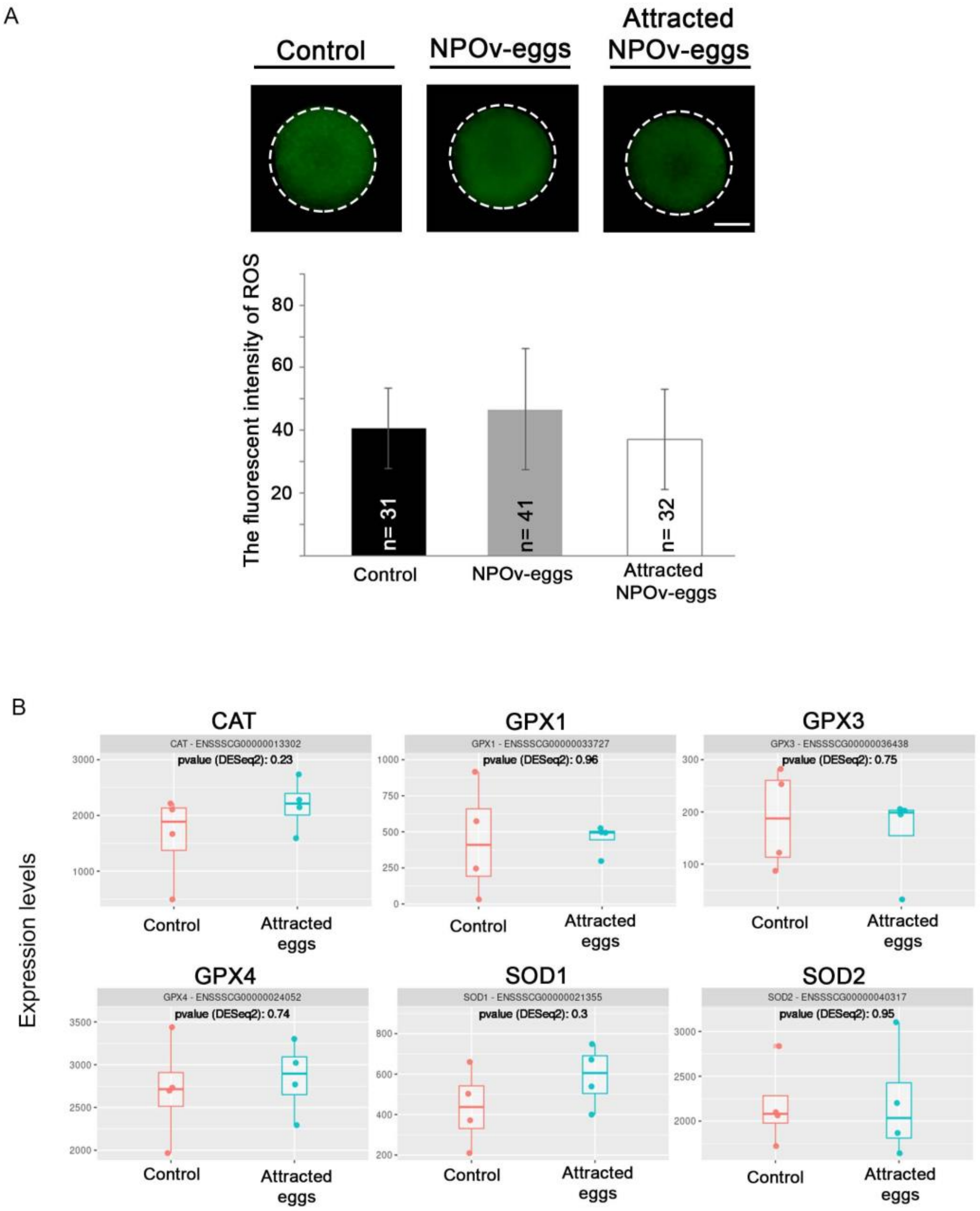

Fig. S4. NPOv and magnetic force do not affect oxidative stress in eggs/embryos. (A) Intracellular reactive oxygen species (ROS) generation in NPOv-eggs. Eggs were co-incubated with NPOv (NPOv-egg group) for 1 h, and a group of these eggs were subjected to a magnetic field (magnet, Cytiva Life SciencesTM MagRack 6, Fisher Scientific) (attracted NPOv-eggs group). For the control group, eggs were absent of NPOv. The generation of reactive oxygen species (ROS) in eggs was evaluated using a DCFDA/H2DCFDA-Cellular ROS Assay Kit. Representative fluorescence microscope images of intracellular ROS (green fluorescence) in living eggs are shown. Bar scale= 50 µm). The oxidative stress was not affected neither for the presence of NPOv nor the magnetic force (Kruskal-Wallis, P> 0.05). Data are presented as mean ± SD (n=3 replicates, a total of 104 eggs). **(B) NPOv-embryos, derived from magnetic attracted eggs, show no changes in gene-related oxidative stress expression.** Based on the transcriptomic analysis (RNA-seq) expression levels of catalase (CAT), glutathione (GPX1, GPX3, GPX4), and superoxide-dismutase (SOD1, SOD2) genes were compared between blastocyst developed from non-attracted eggs (control group) and eggs attracted by a magnetic force (attracted eggs group) (P> 0.05) (n=4 replicates, each replicate was composed by a pool of 10 blastocysts per experimental group).

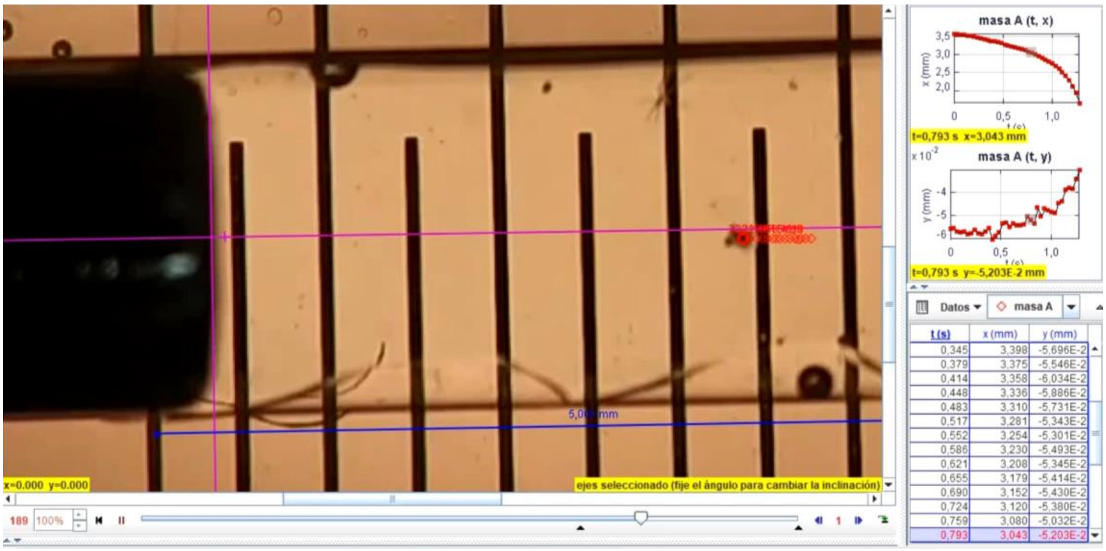

Fig. S5. Video showing an NPOv-egg (red dots) under the influence of a magnetic force (magnet in the left part of the image). In this case, the NPOv-egg was located ~6 mm far from the magnet. The experiment was performed in a modified glass microslides (BTX Microslides^{TM}) covered by PBS media (38.5 ºC). The separation between each vertical line corresponds to 1 mm. The tracking of the NPOv-egg was recorded and evaluated by Tracker 6.0.8 software (https://physlets.org/tracker/).

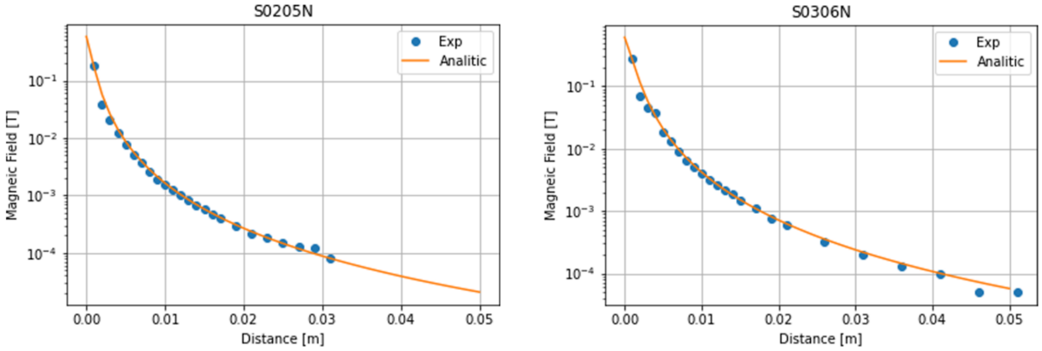

$$B = \frac{\mu_0 M}{2}\left[\frac{z+h}{\sqrt{(z+h)^2+a^2}} - \frac{z}{\sqrt{(z+h)^2+a^2}}\right]$$

Fig. S6. Characterization of the magnets. Axial component of the magnetic field (in T) as a function of the axial distance (in m) produced by S0205N (left) and S0306N (right) magnets. The experimental data (dots) were obtained by using an axial Hall probe (Phywe 13610.01) connected to a Tesla-meter (Phywe 13610.93) and the theoretical data (solid lines) correspond to cylindrical magnets with the same dimension as the experimental ones and a magnetization in the axial *z*-direction: where *h* and *a* are, respectively, the altitude and radius of the magnet, *z* the axial distance to the magnet surface, μ_0 the permeability of the free space and *M* the magnetic dipole moment per unit volume of the magnet. The values obtained for *M* are 10x10^5 A/m (left) and 10.5x10^5 A/m (right) that result to be around 5% smaller than the specifications of the provider for N45 type (between 10.5x10^5 A/m and 10.9x10^5 A/m) and N48 type (between 10.9x10^5 A/m and 11.3x10^5 A/m) NdFeB magnets. The data obtained for S0202N magnet are not shown because the significant experimental errors associated to the small dimensions of this magnet.
